# Do Farm Subsidies Effectively Increase Grain Production? Evidence from Major Grain-Producing Regions of China

**DOI:** 10.3390/foods12071435

**Published:** 2023-03-28

**Authors:** Tingwei Yang, Abbas Ali Chandio, Aopeng Zhang, Yan Liu

**Affiliations:** College of Economics, Sichuan Agricultural University, Chengdu 611130, China; 202003166@stu.sicau.edu.cn (T.Y.); alichandio@sicau.edu.cn (A.A.C.); 202001639@stu.sicau.edu.cn (A.Z.)

**Keywords:** agricultural subsidies, grain production, food security, China

## Abstract

The purpose of this paper is to investigate the impact of agricultural subsidies on grain production in major grain-producing regions. We use an empirical model and data from fixed observation points in rural areas collected by the Ministry of Agriculture and Rural Affairs in 2016–2017. Our findings show that agricultural subsidies in major grain-producing regions have significantly increased rural household grain yield. Furthermore, findings show that agricultural subsidies and the cost of fertilizer and pesticides for rural households have a mediating effect on grain production. In addition, the effect of agricultural subsidies varies by type: income subsidies have a greater promotion effect on grain production, whereas subsidies for purchasing agricultural machinery have no significant promotion effect on grain production. These findings show that agricultural subsidies promote grain production in China’s major grain-producing regions, and have a mediating effect on different types of subsidies.

## 1. Introduction

Globally, food insecurity is a major issue. Since 2022, the endurance of the Russia–Ukraine conflict has amplified global uncertainty and posed new challenges [1,2]. Food security has been widely debated in recent years [3,4,5,6,7], and it is a key area of research that has broadly attracted both domestic and international experts and created a wealth of research results. As one of the primary components of food security, grain production has also been determined to be essential for food security. Additionally, scholars have discovered that grain production is essential for food security [8]. Therefore, food security is a crucial issue in China and it has been frequently mentioned since 2022. To ensure national food security in 2022, the No. 1 central document emphasized the need to “firmly hold the bottom line of food security” and “deepen the implementation of the quality food project” [8,9].

To ensure food security, focusing on grain production is imperative [5,10]. According to the United Nations Food and Agriculture Organization, the goal of food security is to “ensure that all people have access to and can afford the basic food they need at all times” [11]. Due to the close link between food security and grain production, the No. 1 central document put the command of stabilizing sown areas and grain production first, which shows the significance of grain production for food security [8,9]. In addition, in November 2022, the central government advanced CNY 21.5 billion in agricultural transfer payments for 2023 to guarantee grain production.

Agricultural subsidy is a crucial component of grain production that ensures efficient grain production [12,13,14]. China is an agricultural nation similar to Bhutan, and, hence, it prioritizes agricultural subsidies in its agricultural policy to encourage agricultural productivity, raise the income of rural citizens, and increase food security [15]. Agricultural subsidies are also linked to farming and rural residents. On the one hand, rural residents are the backbone of grain production, and agricultural subsidy policy is a vital tool for the government to ensure agricultural workers’ living standards and food security, as well as to increase agricultural residents’ overall income [16]. On the other hand, regions where agricultural subsidies have been eliminated, which produce grain as part of agricultural development, have been studied, and it was discovered [17] that the elimination of subsidies had negative effects on the local non-agricultural economy. A number of studies have, however, concluded that agricultural subsidies in the EU must be drastically decreased [18]. The favorable effects of agricultural subsidies have been found to be most pronounced in major grain-producing regions, where grain is widely harvested [19,20]. Some studies also indicate that the federal government should subsidize grain-producing regions significantly [21]. 

Hence, several studies have investigated the relationship between agricultural subsidies and grain production in various parts of the world. Studies have found (in the literature) that agricultural incentives affect agricultural production [22]. Celik [23,24] concluded that agricultural subsidies could be a significant element influencing grain production. In addition, it has been demonstrated that if the rate of technological growth is more sensitive to changes in land productivity protection subsidy policies than the rate of consumption growth, subsidy policies can be detrimental to long-term growth when the equilibrium growth path is determined [25].

Specifically, there are numerous types of agricultural subsidies, and certain types of subsidies have been introduced since China eliminated the agricultural tax in 2006 [26]. According to their role in grain production, there are now several key subsidies [27,28]. The first is the income-based subsidy, in which the subsidy is given directly to rural people in order to increase their income. As a major grain-producing region, Sichuan provides CNY 324 million in subsidies to its successful growers of grain in order to stimulate grain output. The second is subsidies for the purchase of agricultural machinery, which are issued separately by the Ministry of Finance in the “Guidelines for the Implementation of Subsidies for the Purchase of Agricultural Machinery from 2021 to 2023” [29]. Finally, there are the general subsidies for agricultural supplies, which focus on the security of fertilizers, insecticides, and other materials necessary for grain production. In 2022, the central government allocated CNY 40 billion in three tranches for one-time agriculture subsidies [30]. 

In addition, specifically in China, a number of provinces have implemented policies to encourage and support grain production. Jilin Province, a key grain-producing region, has given CNY 1.07 billion in agricultural subsidies for grain production. The 20th national conference of the Communist Party of China emphasized the need to enhance the mechanism for assuring rural households’ income from grain and the mechanism for compensating large grain-producing regions. In terms of policies, the Government Work Report for 2022 mentions “growing support for main producing sectors” [31].

Furthermore, scholars have focused on how agricultural subsidies affect grain production. Using panel data from 2000–2014, Meng [20] demonstrated that agricultural subsidies had a considerable favorable effect on rural families’ willingness to scale management, with this positive effect being most obvious in main grain-producing regions. Meanwhile, a study in the United States found that the distribution of agricultural subsidies suggests that as farm size becomes smaller, subsidies become more important and may play a role in slowing farm size contraction [32]. In particular, the riskiness of grain production influences the scale of grain cultivation. It is possible that the risk-reducing effect of agricultural subsidies on the low- and middle-income classes is statistically significant, and that agricultural subsidies would then indirectly affect the volume of grain production by affecting the scale of cultivation [33]. The willingness to grow grain is likewise an important mechanism affecting the effectiveness of agricultural subsidy policies [34], and reducing the risk of growing grain may increase the willingness to grow and incentivize rural households to expand the scale of cultivation. However, at the same time, this effect is not thought to be identical across recipients, and there are differences in the effects of agricultural subsidy policies on a rural households’ sown area [35]. Other studies have examined the effect of subsidized agriculture on crop sown areas, concluding that subsidies have both stimulating and inhibiting effects [36].

In terms of production efficiency, the China Agricultural Mechanization Promotion Law and the agricultural machinery purchase subsidy policy have been implemented since 1998 and 2004, respectively. The policy and the law’s goal is to increase agricultural mechanization in China [37]. It is possible that an increase in budgetary funds, i.e., a policy of direct subsidies for machinery purchases, will significantly improve agricultural production. According to Bagheri [38], labor is the primary driver of wheat production growth, and agricultural machinery can be an effective labor substitute in grain production. The study’s findings also revealed that rural households regard changes in farming practices and farm machinery sharing as important risk management strategies. The study by Kugbadzor and Ganbold [39,40] found that, on average, EU Common Agricultural Policy (CAP) subsidies promote agricultural labor productivity growth, but this combined effect hides important heterogeneity in the effects of different types of subsidies. It also means that there are differences in the effects of different types of subsidies [41]. This is what this paper wants to discuss specifically.

Regarding fertilizer application, Kugbadzor [39] evaluated the influence of the Fertilizer Subsidy Program (FSP) on agricultural output in Ghana. The results indicated that the national output elasticity increased when the FSP was implemented [42]. The study indicated that agricultural subsidies helped rural households minimize their use of fertilizer [43]. However, the pattern of fertilizer application also influences grain yield, and one study [44] revealed that agricultural subsidies were beneficial for increasing the use of green insecticides. According to a study of Qian [45], a 1% increase in agricultural subsidies reduces fertilizer use by an average of 3.4%, based on survey data from 2014 to 2018. According to the findings of a study based on farm-level data in Africa, fertilizer subsidy programs appear to be effective tools to increase farm efficiency [46].

The goal of this paper is to investigate how agricultural subsidies affect grain production in major grain-producing regions. More specifically, the marginal contributions of this paper are concentrated in three areas: (1) The existing literature contains less analysis of the various effects of various types of agricultural subsidies, and this paper supplements the existing literature on the margin. (2) There are few researchers who have analyzed specific types of subsidies, and this paper focuses on which types of agricultural subsidy are more efficient in order to develop the recommendation of targeted policy. (3) This paper uses micro data to specifically observe the effects of agricultural subsidies on rural households’ grain production based on the basic situation of small rural households in a large country such as China. (4) This paper uses mediating effect model and select off-farm employment as one of the mediating variables.

The rest of the paper is organized as follows. Section 2 deals with the theoretical framework, while Section 3 deals with the methodology and data. Section 4 gives the empirical results and discussion. Section 5 contains the conclusion and policy recommendations.

## 2. Theoretical Analysis and Research Hypotheses

Agricultural subsidy plays an essential role in ensuring and promoting grain production and further enhancing food security [47,48,49]. Therefore, this paper analyzes the agricultural subsidies affecting grain production in major grain-producing areas. Agricultural subsidies can increase households’ income [15,50], as they can stimulate the expansion of acreage and promote grain production. Persistently, the current main force in China’s grain production is small households [51,52,53,54]. Therefore, generating enthusiasm in rural households for grain production contributes significantly to producing grain and ensuring food security. Hence, the government distributes subsidies directly to households cultivating grains in major producing areas. It is classified as an income-based subsidy, increasing households’ income directly [55,56]. Thus, income-based subsidy is an effective way to incentivize grain production [57,58,59]. Since the income-based subsidy is distributed to grain-producing areas, on the one hand, the more extensive acreage they cultivate, the more subsidies they obtain, which will stimulate households to have their acreage enlarged [57,58,60,61]. On the other hand, the income from grain production becomes increased. For rural households, especially those in the major grain-producing areas, producing grain is a primary resource of income [62,63,64]. There is a possibility that acreage will be increased to receive higher subsidies, in which case the production of grain is promoted and the income sale of grain consequently receives a boost. Furthermore, it stimulates rural households to cultivate grain. Therefore, income-based subsidies contribute significantly to enlarging acreage and increasing grain production. 

H1: Income-based subsidy significantly improves grain output in the major grain-producing area of China.

Agricultural machinery and equipment purchase subsidies (farm machinery subsidies) are financial subsidies provided by the central and local governments that encourage households to have agricultural machinery advances [65]. Thus, this subsidy increases the degree of agricultural mechanization and improves agricultural productivity [36,66,67]. First, for rural households, one of the benefits is that subsidizing the purchase of agricultural machinery will improve the quantity and quality of machinery [68,69,70] that rural households purchase. With agricultural machines, they can input less labor per acre and produce grain more efficiently [40,71,72]. Another advantage is that agricultural machinery can help alleviate agricultural disasters and function in different places [73,74,75]. Advanced machinery puts less constraints on time and space, and, thus, improves production efficiency. The second advantage mainly concerns grain production. With updates in agricultural machinery, machines will be the mediators for more advanced and greener agricultural technology. For example, machinery, such as subsoilers, can loosen the soil and improve soil fertility [76,77], build irrigation and water conservancy systems, and control diseases and pests. These agricultural technologies will not only reduce errors caused by human activities but also improve grain production efficiency and promote grain production in the long term.

H2: Agricultural subsidies can increase agricultural mechanization, thereby increasing production efficiency and further promoting grain production.

Comprehensive subsidies for agricultural supplies are mainly related to the inputs of grain production [65], such as fertilizers and pesticides. As we all know, agricultural subsidies can cover part of the expense of rural households in major producing areas. The increase in the budget for fertilizer and pesticides will result in changes in households’ usage patterns [21,42,78,79]. Without subsidies, the rural households that produce grain have to pay for the expense of fertilizer and pesticides. As a result, they tend to purchase cheap fertilizers or mono-compound fertilizers [80,81], resulting in poor soil quality and polluted water resources [82]. However, if the government allocates subsidies for agricultural supplies to households, they will possibly change their choice. For sensible rural households, they want to maximize the expected rate of return or discount the predicted rate of return. Due to the small acreage most rural households cultivate, applying fertilizers and pesticides is an efficient way to manage the risks of grain production and uncertain weather [83]. Subsidies to agricultural supplies are crucial to improve fertilizer application decisions [84,85,86]. Subsidies enable rural households to access more effective fertilizers and mitigate soil degradation. This improves soil quality and ensures sustainable land development in key growing areas [87,88], further boosting grain production in the long run.

H3: Agricultural subsidies improve fertilizer and pesticide application behavior, enhance land quality, and boost grain production.

## 3. Data and Methodology

### 3.1. Data 

**Figure 1:**
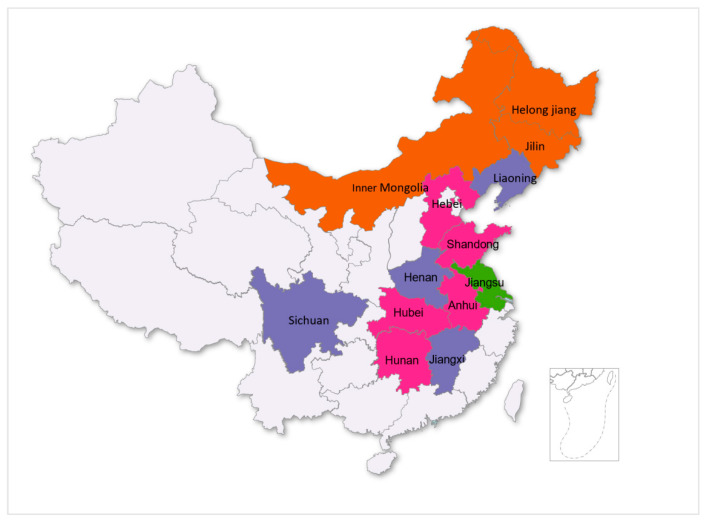
Map of the study area.

**Figure 2:**
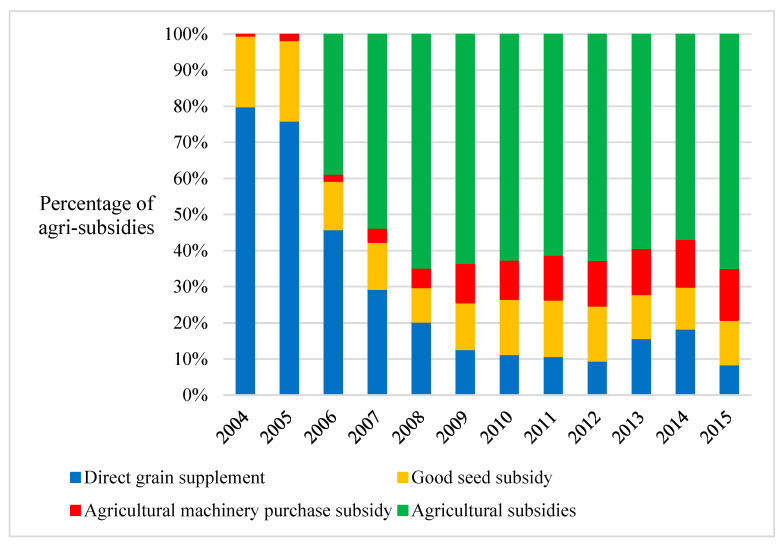
Different types of agricultural subsidies from 2004–2015. Note: Direct grain supplement: a subsidy paid directly to farmers who grow grain to increase their income. Good seed subsidy: a subsidy paid to food farmers to support the use of better crop seeds. Agricultural machinery purchase subsidy: a subsidy given to grain-growing farmers for the purchase and upgrade of agricultural machinery for grain production. Agricultural subsidies: a direct subsidy given to farmers who grow food to purchase the means of agricultural production, including fertilizer, diesel fuel, seeds, and farm machinery.

This paper analyzes data from the Ministry of Agriculture and Rural Affairs’ National Rural Fixed Observation Point Survey. We use cross-sectional survey data from 2017 based on data availability and study needs. The scope of this paper was based on the 13 major grain-producing provinces (see Figure 1) in China. Crop cultivation is closely related to the geography of the province where rural households are located and is influenced by certain geographical characteristics; rural households in the same province may choose to rotate wheat and maize, rice and maize, or staple grains with other crops. After excluding samples with outliers (values that deviate from the sample mean by a greater or lesser amount) and missing values, as well as samples from villages with the insufficient sample size of 10 households, a final sample of 3472 rural households was used in this paper’s analysis. Table 1 contains descriptive statistics for the variables studied, while Figure 2 depicts the trend of various types of agricultural subsidies.

1. Explanatory variables

The main food crops in China are rice, wheat, maize, and soybeans, and the sum of their yields is used to measure household food production.

2. Core explanatory variables

Agricultural subsidies are measured by the number of agricultural subsidies received by farmers, including: direct subsidies for grain, subsidies for good seeds, comprehensive subsidies for the purchase of production materials, and subsidies for the purchase and renewal of large agricultural machinery.

3. Mediating variables

The cost of machinery operations, fertilizer inputs, and pesticide inputs in food cultivation are used as mediating variables.

4. Control variables

The control variables in the model includes the following categories. ① Demographic characteristics, including the age, gender, education level, and ethnicity of the household head. ② Household characteristics, including the number of household laborers; whether the household is a party member; the annual net income of the household; the area of cultivated land; the fixed assets of household facilities for agriculture; and the cost of food, water, electricity, and irrigation. ③ Village characteristics, including topography and village water conservancy facilities. Specific descriptions are shown in Table 1.

### 3.2. Empirical Model

To investigate the impact of agricultural subsidies on grain production, the following model was developed for estimation:

(1) $${\mathrm{Grain}}_{i}\;=\;\alpha_{0}\;+\alpha_{1}X_{i}\;+\;\alpha_{2}Z_{i}\;+\;\mu_{i}$$

where is the explanatory variable; denotes the coefficient to be estimated; *i* denotes the micro-individual; X_i_ denotes the agricultural subsidy; Z_i_ denotes other control variables, including individual characteristics, household characteristics, production and operation characteristics, village characteristics, etc.; and *μ*_i_ denotes the random error term of model (1).

To further test the mechanism of agricultural subsidies affecting grain yield, the mediating variables including machinery operation cost, fertilizer input, and pesticide input were added to the basic model (1). The mediating effect model is developed as follows:


(2) $${\mathrm{Grain}}_{i}\;=\;\alpha_{0}\;+\alpha_{1}X_{i}\;+\;\alpha_{2}Z_{i}\;+\;\varepsilon_{i}$$

(3) $${\mathrm{Agrinput}}_{i}\;=\;\beta_{0}\;+\beta_{1}X_{i}\;+\;\beta_{2}Z_{i}\;+\;\varepsilon_{i}$$

(4) $${\mathrm{Grain}}_{i}\;=\;\theta_{0}\;+\theta_{1}{\mathrm{Agrinput}}_{i}\;+\;\theta_{2}X_{i}\;+\;\theta_{2}Z_{i}\;+\;\varepsilon_{i}$$

In this paper, Grain_i_ is the dependent variable, which denotes the grain yield of the unobservable sample households. In addition, X_i_ denotes the explanatory variables; Agrinput_i_ denotes the mediating variables such as agricultural production inputs; α, β, and θ denote the parameters to be estimated; Z_i_ denotes the control variables; and ε_i_ denotes the residual term, which measures a series of unobservable factors.

In the model presented above, agricultural subsidies are the primary explanatory variable, grain yield is a dependent variable, and machinery operating expenses, fertilizer input, and pesticide input are the mediating factors. Since agricultural subsidies are implemented by the government through laws and policies, meaning that such variables are exogenously given, endogeneity due to sample self-selection can be excluded; however, the model may have endogeneity due to omitted variables, and this paper employs the control variables method in order to control as many potentially influential variables as possible. We include control variables such as personal characteristics, household characteristics, agricultural production characteristics, and village features in order to reduce the bias resulting from omitted variables.

#### Selection of Model Variables

(1) Dependent variable: rice, wheat, corn, and soybeans are the most important grain crops grown in China. The sum of grain crop yield is used in this paper to calculate farm household grain production.
(2) Independent variables: a number of agricultural subsidies received by rural households, including direct grain subsidies, subsidies for high-quality seeds, all-inclusive subsidies for buying production materials, and subsidies for buying and upgrading large agricultural machinery.
(3) Mediating variables: the cost of machinery operation, fertilizer input, and pesticide input in grain cultivation.
(4) Control variables: The control variables in the model included the following categories. ① Demographic characteristics, including the age, gender, education level, and ethnicity of the household head. ② Household characteristics, including the number of the household labor force; whether the household is a party member; the annual net income of the household; the area of cultivated land; household facilities and agricultural fixed assets; and the cost of water, electricity, and irrigation for food. ③ Village characteristics, including terrain and village water conservancy facilities.

## 4. Results and Discussion

### 4.1. Baseline Regression

In Table 2, Column (1), the estimation results are shown without the inclusion of control variables, and the results show that agricultural subsidies have a significant positive effect at the 1% level, with a coefficient of about 0.621, and that agricultural subsidies contribute to grain production. The yield-increasing effect of agricultural subsidies decreases after gradually including control variables for individual characteristics, household characteristics, and village characteristics in Columns (2), (3), and (4), but it remains significantly positive at the 1% level, indicating the existence of the effect of the control variables.

Column (1) is the estimated result without the inclusion of control variables. Agricultural subsidies have a significant positive effect at the 1% level, with a coefficient of about 0.621, and have a positive contribution to grain production. Columns (2), (3), and (4) gradually add the control variables of individual characteristics, household characteristics, and village characteristics, and, with these additions, the yield-increasing effect of agricultural subsidies decreases, but is still significantly positive at the 1% level, indicating that the effect of the control variables exists.

Furthermore, the effects of control variables on food production are as follows:

① The effect of individual characteristics variables: At the 1% level, ethnicity has a positive and significant effect on household grain production, whereas age, gender, and education level have no effect. It is observed that Han ethnicity households have more education and knowledge of modern agriculture than ethnic minority households, and ethnic minorities mostly live in remote areas with harsher climatic and terrain conditions. Hence, Han ethnicity households will have higher grain production yields.

② The effect of household characteristics on grain production: Arable land area, food utilities and irrigation costs, and annual household net income can considerably contribute to an increase in grain yield at the 1% significance level. Arable land is directly related to grain production, and households with more arable land will have more land resources for grain cultivation, thereby promoting grain production and income growth to a certain extent. The process of grain crop cultivation and production requires suitable water and irrigation conditions, and households with higher investments in water and electricity irrigation will have more substantial irrigation resources, thereby promoting grain production and income growth. The influence of household income on their decision-making behavior is substantial. To increase the size of grain production, purchase high-quality grain seeds, and enhance planting conditions, etc., households require specific financial assistance. Households with a higher income might allocate a larger budget to the advancement of grain planting technologies in order to attain the desired high quality and high yield.

③ The impact of village characteristics on grain yield: Crop cultivation has specific requirements for soil quality and topography. An environment with a flatter terrain and more fertile soil has superior conditions for grain cultivation; an environment with steep terrain and a poor climatic environment will negatively affect grain crop growth and, to some extent, prevent normal grain production.

### 4.2. Mediating Effects

#### 4.2.1. Mediating Effects of Mechanization

In general, we believe that increased fertilizer and pesticide use and the adoption of mechanized production methods will boost food production, whereas rural families with less fertilizer and pesticide inputs and less mechanization will produce less food. On the one hand, agricultural subsidies raise the capital of rural families for agricultural output and offset the price of advancing agricultural technologies. Agricultural subsidies, on the other hand, fundamentally alter rural households’ attitudes toward advanced production technologies and agricultural production inputs, which in turn influences rural households’ input decisions regarding factors of production, such as pesticides, fertilizers, and the adoption of mechanized farming technologies. Consequently, this paper further investigates this fundamental mechanism based on the baseline regression.

As shown in Table 3, the estimated coefficients of agricultural subsidies on machinery operation and grain yield are significantly positive. Mechanical activity was an intermediate variable, and the coefficient of the total effect of agricultural subsidies on grain output was 0.527. In addition, after adding the mechanical operation cost, the influence coefficient θ_1_ of rural households’ mechanical operation cost on grain yield is 0.564, which is significant at a 1% level, and the coefficient θ_2_ of agricultural subsidies on grain yield is still significantly positive.

After receiving agricultural subsidies, rural households change their production and planting practices, so that agricultural production decisions tend to mechanize operations and improve the efficiency of crop planting and cultivation; thus, these households distribute some of the rural households’ agricultural production pressure. Additionally, rural households have more energy to spend on learning advanced planting experience, and learning how to improve food cultivation and management and increase food production.

#### 4.2.2. Pesticide Input Mediating Effect

Table 4 demonstrates the mediating impact between pesticide input and grain yield of rural families engaged in grain production, and the influence coefficients of agricultural subsidies on both pesticide input and grain yield are significantly positive. Next, we included the variable pesticide input. The influence coefficient of pesticide input by rural household on grain yield was 0.316, which was significant at the 1% level. The influence coefficient of agricultural subsidies on grain production remained strongly positive, but at a lower level. This suggests that pesticide input partially mediates the relationship between agricultural subsidies and grain yield. In particular, 3% of the influence of agricultural subsidies on grain yield is attributable to the use of pesticides in grain sowing.

The Chinese government has implemented a number of supportive measures, including direct grain subsidies, general subsidies for agricultural goods, premium subsidies for agricultural insurance, and natural disaster relief subsidies. As a policy distinct from price support, agricultural subsidies have a clear synergistic impact, in that they can reduce production costs and enhance rural families’ net income from grain farming more effectively, thereby raising rural households’ enthusiasm for grain farming.

Presently, China’s food security is currently in jeopardy due to dwindling arable land per capita, rising environmental strain, and rising grain demand. The use of pesticides has a favorable impact on controlling crop diseases and insect pests and boosting output, but it has a detrimental impact on soil quality and sustainable soil growth. Increasing the technological research and development of pesticides, supporting the development of sustainable agriculture, and enhancing the utilization efficiency of pesticides are steps that are crucial to successfully minimizing environmental pollution and ensuring grain production.

#### 4.2.3. Mediating Effect of Fertilizer Use

As shown in Table 5, in Equation (1), the coefficient of agricultural subsidies is 0.527, and, in Equation (2), the coefficient of agricultural subsidies is 0.453 and significant at the 1% level, indicating that agricultural subsidies have a significant positive effect on fertilizer input. In addition, the coefficients of agricultural subsidies and fertilizer input in Equation (3) are 0.141 and 0.852, respectively, both of which are significant, indicating that agricultural subsidies and fertilizer input have a significant promoting effect on grain output. Through the Sobel–Goodman mediation effect test, it is concluded that the mediation effect accounts for 73.2%. It shows that, while agricultural subsidies promote grain yield, fertilizer input also promotes the effect of agricultural subsidies on grain yield to a certain extent.

Agricultural subsidies have certain positive externalities on agricultural development by influencing rural households’ fertilizer input behavior. As one of the production factors of modern agriculture, fertilizer provides nutrients to soil for crop growth and plays an important role in the improvement of grain yield. Under the background of less land and more people, fertilizer is the primary means with which to meet the needs of food production. Increasing the utilization rate of chemical fertilizer and the proportion of organic fertilizer application; improving soil fertility; ensuring soil quality and sustainable development; and seeking an efficient, safe, and environmentally friendly modern agricultural development road will further promote grain yield and income.

### 4.3. Heterogeneity Analysis

It can be concluded from (1), (2), and (3) in Table 6 that income subsidies have the greatest impact on grain production, with a coefficient of 0.539, and are significant at the 1% level. The coefficients of subsidies for improved varieties and comprehensive subsidies for purchasing means of production are 0.485 and 0.321, respectively, and are still significant at the 1% level. It can be concluded from (4) in Table 7 that the purchase and renewal of large-scale agricultural machinery subsidies have no significant impact on grain output. In terms of agricultural subsidies, the government has increased direct grain subsidies and subsidies for improved seed varieties to households, has increased households’ enthusiasm for grain growing, has promoted the popularization of scientific and efficient grain growing technologies, and has organically integrated various kinds of subsidies, which will help better promote grain yield and income, in order to ensure the realization of food security goals to some extent.

### 4.4. Robustness Test

1. Replacement with 2016 data

To ensure the robustness of the regression results, the data of 2016 are used in this paper for re-regression, and 3423 samples are obtained after processing. Through the analysis of the regression results of column (1) in Table 7, the symbols and significance of the main explanatory variables have not changed substantially, indicating that the above regression results are robust.

2. Tail reduction treatment

To improve the rigor of the research logic, this paper re-estimates the impact of agricultural subsidies on grain output by using tail reduction processing on sample data. The estimated results show that agricultural subsidies significantly promoted rural households’ grain yield increase, which increased the probability of rural households’ grain yield increase by 44%, again verifying the robustness of the baseline regression results.

## 5. Conclusions and Policy Recommendations

This study aims to explore the impact of agricultural subsidies on grain production. It was proven that agricultural subsidies significantly and positively contribute to the growth of grain production in major producing areas of China. The study found that, all else being equal, each unit increase in the number of agricultural subsidies distributed to households in the major producing areas increased the average grain production of the households by 0.527%. Agricultural subsidies can be classified as income-based subsidies, subsidies for agricultural supplies, and subsidies for the purchase of agricultural machinery and tools. For each unit of income-based subsidy, this study found that the grain yield of households in Sichuan increased by 0.539%. From the perspective of agricultural supplies such as fertilizer and pesticides, the findings of the analysis showed that 3% of the impact of agricultural subsidies on grain yield was due to pesticide input in grain planting, and the influence coefficient of fertilizer input on grain yield was 0.852. From the perspective of production efficiency, this study found that after adding the intermediate variable of machinery cost, the influence coefficient of rural households’ machinery operation cost on grain yield was 0.564. In addition, the intermediate effect accounted for 25.1% of the promoting effect of agricultural subsidies on grain yield.

To investigate the promotion of grain production in the major grain-producing regions, this study combined the current policies and its results. On one hand, planting areas needs to be increased; a larger planting area can ensure grain production. On the other hand, agricultural mechanization is of great importance, since technology is the trend of modern agriculture. Lastly, the behavior of fertilizer use has a long-run impact on land quality as well grain production.

To place some recommendations for the policies that can promote grain production, we analyze the empirical findings. Enhancing the main grain production in the major producing area of Sichuan province, in order to ensure and improve food security in China, is the recommendations’ primary concern.

First, we recommend that the government increases subsidies in a targeted way, since the transfer income the government can offer is limited. Hence, increasing a type of subsidy that is more efficient in promoting grain production and maximizing the effect of the subsidy is of prime importance. Second, because the current price level of grains is not high, and because farming is not very profitable and the cost of planting is rising year after year, increasing the income-based subsidy is essential. Increasing income directly promotes grain production; therefore, the income-based subsidy should be adjusted every year to ensure that rural households can get the income they are satisfied with.

Third, mechanization also plays a significant role in improving production efficiency and increasing grain production; thus, the universal usage of agricultural machines should be vigorously promoted, and the amount of machines put into use needs to be increased.

Finally, we recommend an increase to subsidies for fertilizers and pesticides to improve the quality of land farm grains. It is known to all that fertilizers and pesticides can increase the production of grains, but, currently, the excessive use of fertilizers and pesticides can cause the degradation of land quality and, even worse, agricultural non-point source pollution.

## Figures and Tables

**Table 1:** Descriptive statistics.

Variables	Symbol	Variable Definitions	Mean	SD
Grain yield	Grain output	The logarithm of grain yield	7.79	0.996
Agricultural Subsidy	Subsidy	The logarithm of agricultural subsidy (CNY)	6.30	0.951
Fertilizer input cost	Fer	The logarithm of fertilizer cost (CNY)	6.53	0.911
Pesticide input cost	Pesticide	The logarithm of pesticide cost (CNY)	4.23	0.431
Mechanical operation expense	Mech	The logarithm of mechanical operation expense (CNY)	6.34	0.805
Gender	Gender	Male = 1, Female = 0	1.08	0.274
Age	Age	Year	57.53	11.803
Length of Education	Edu	Length of education (Year)	7.08	2.577
Household with party member	Dy	Yes = 1, No = 0	0.16	0.370
Ethnicity	Nation	Han ethnicity = 1, Ethnic minorities = 0	0.88	0.324
Household labor force	Labor	The number of participating in labor in households (Person)	2.40	1.208
Cultivated area	Size	Cultivated area (Mu)	9.29	19.644
Household facilities of agricultural fixed assets	Fixed	The logarithm of agricultural fixed assets (CNY)	7.92	0.394
Water, electricity, and irrigation costs for grain	Irrigate	The logarithm of water, electricity, and irrigation costs for grain (CNY)	5.33	0.610
Annual household net income	Income	Household net income (CNY)	10.68	0.938
Terrain	Land		1.84	0.795
Village water conservancy facilities	Hydraulic	The logarithm of village water conservancy facilities cost (CNY)	7.50	1.427

**Table 2:** Estimated results of the impact of agricultural subsidies on food production.

	(1)	(2)	(3)	(4)
	lnGrain Yield	lnGrain Yield	lnGrain Yield	lnGrain Yield
lnAgricultural subsidy	0.621 ***	0.616 ***	0.521 ***	0.527 ***
	(47.154)	(46.504)	(37.307)	(37.845)
Gender		0.008	−0.018	−0.007
		(0.141)	(−0.314)	(−0.123)
Age		−0.004 ***	−0.002	−0.002
		(−3.053)	(−1.280)	(−1.297)
Length of education		0.007	0.007	0.007
		(1.133)	(1.086)	(1.218)
Ethnicity		0.296 ***	0.281 ***	0.262 ***
		(5.010)	(5.020)	(4.692)
Household with party member			0.027	0.023
			(0.727)	(0.619)
Household labor force			−0.003	−0.002
			(−0.256)	(−0.136)
Cultivated area			0.005 ***	0.005 ***
			(8.607)	(8.879)
lnHousehold facilities of agricultural fixed assets			−0.054	−0.048
			(−1.526)	(−1.343)
lnWater, electricity and irrigation costs for grain			0.354 ***	0.351 ***
			(17.627)	(17.555)
lnAnnual household net income			0.084 ***	0.078 ***
			(4.185)	(3.933)
Terrain				−0.104 ***
				(−5.869)
Village water conservancy facilities				−0.000
				(−0.528)
_Cons	3.964 ***	3.900 ***	2.029 ***	2.197 ***
	(45.437)	(23.323)	(5.061)	(5.481)
N	3472	3472	3472	3472
r2	0.391	0.397	0.466	0.471
r2_a	0.390	0.396	0.464	0.469

Note: *** indicate significance levels at 1%, respectively.

**Table 3:** Mediating effects of mechanization.

	(1)	(2)	(3)
	lnGrain Yield	lnMechanical Operation Expense	lnGrain Yield
lnAgricultural subsidy	0.527 ***	0.235 ***	0.394 ***
	(37.845)	(18.593)	(31.370)
lnMechanical operation expense			0.564 ***
			(35.026)
Age	−0.002	−0.002	−0.001
	(−1.297)	(−1.590)	(−0.581)
Length of education	0.007	−0.003	0.009 *
	(1.218)	(−0.503)	(1.711)
Household with party member	0.023	0.013	0.016
	(0.619)	(0.380)	(0.498)
Ethnicity	0.262 ***	0.294 ***	0.099 **
	(4.692)	(5.810)	(2.053)
Household labor force	−0.002	0.004	−0.003
	(−0.136)	(0.355)	(−0.298)
Cultivated area	0.005 ***	0.005 ***	0.003 ***
	(8.879)	(8.714)	(5.117)
lnHousehold facilities of agricultural fixed assets	−0.048	0.006	−0.051 *
	(−1.343)	(0.194)	(−1.686)
lnWater, electricity and irrigation costs for grain	0.351 ***	0.361 ***	0.147 ***
	(17.555)	(19.930)	(8.096)
lnAnnual household net income	0.078 ***	0.095 ***	0.024
	(3.933)	(5.245)	(1.394)
Terrain	−0.104 ***	−0.140 ***	−0.025 *
	(−5.869)	(−8.742)	(−1.674)
Village water conservancy facilities	−0.000	−0.000	
	(−0.528)	(−1.159)	
lnVillage water conservancy facilities			−0.008
			(−0.933)
_Cons	2.197 ***	1.988 ***	1.150 ***
	(5.481)	(5.463)	(3.255)
N	3472	3472	3472
r2	0.471	0.306	0.610
r2_a	0.469	0.303	0.608

Note: *, **, and *** indicate significance levels at 10%, 5%, and 1%, respectively.

**Table 4:** Mediating effect of pesticide input.

	(1)	(2)	(3)
	lnGrain Yield	lnPesticide Input Cost	lnGrain Yield
lnAgricultural subsidy	0.527 ***	0.049 ***	0.511 ***
	(37.845)	(5.669)	(37.120)
lnPesticide input cost			0.316 ***
			(11.724)
Age	−0.002	0.001 *	−0.002 *
	(−1.297)	(1.794)	(−1.684)
Length of education	0.007	−0.003	0.008
	(1.218)	(−0.695)	(1.393)
Household with party member	0.023	−0.001	0.024
	(0.619)	(−0.031)	(0.652)
Ethnicity	0.262 ***	0.008	0.265 ***
	(4.692)	(0.218)	(4.839)
Household labor force	−0.002	0.025 ***	−0.008
	(−0.136)	(3.077)	(−0.610)
Cultivated area	0.005 ***	−0.001	0.005 ***
	(8.879)	(−1.607)	(9.433)
lnHousehold facilities of agricultural fixed assets	−0.048	−0.009	−0.046
	(−1.343)	(−0.408)	(−1.310)
lnWater, electricity, and irrigation costs for grain	0.351 ***	0.119 ***	0.313 ***
	(17.555)	(9.603)	(15.743)
lnAnnual household net income	0.078 ***	0.033 ***	0.066 ***
	(3.933)	(2.702)	(3.374)
Terrain	−0.104 ***	−0.043 ***	−0.087 ***
	(−5.869)	(−3.910)	(−5.169)
Village water conservancy facilities	−0.000	0.000	
	(−0.528)	(0.659)	
lnVillage water conservancy facilities			−0.018 **
			(−1.975)
_Cons	2.197 ***	3.025 ***	1.396 ***
	(5.481)	(12.179)	(3.412)
N	3472	3472	3472
r2	0.471	0.055	0.492
r2_a	0.469	0.052	0.490

Note: *, **, and *** indicate significance levels at 10%, 5%, and 1%, respectively.

**Table 5:** Mediating effects of fertilizer use.

	(1)	(2)	(3)
	lnGrain Yield	lnFertilizer Input Cost	lnGrain Yield
lnAgricultural subsidy	0.527 ***	0.453 ***	0.141 ***
	(37.845)	(34.010)	(15.105)
lnFertilizer input cost			0.852 ***
			(82.625)
Age	−0.002	−0.001	−0.001
	(−1.297)	(−0.799)	(−1.107)
Length of Education	0.007	0.013 **	−0.004
	(1.218)	(2.223)	(−1.015)
Household with party member	0.023	0.024	0.003
	(0.619)	(0.662)	(0.142)
Ethnicity	0.262 ***	0.273 ***	0.030
	(4.692)	(5.116)	(0.908)
Household labor force	−0.002	0.013	−0.012
	(−0.136)	(1.024)	(−1.645)
Cultivated area	0.005 ***	0.005 ***	0.001 ***
	(8.879)	(8.749)	(2.998)
lnHousehold facilities of agricultural fixed assets	−0.048	−0.038	−0.015
	(−1.343)	(−1.133)	(−0.729)
lnWater, electricity, and irrigation costs for grain	0.351 ***	0.330 ***	0.070 ***
	(17.555)	(17.251)	(5.787)
lnAnnual household net income	0.078 ***	0.051 ***	0.035 ***
	(3.933)	(2.680)	(3.001)
Terrain	−0.104 ***	−0.088 ***	−0.028 ***
	(−5.869)	(−5.201)	(−2.813)
Village water conservancy facilities	−0.000	−0.000	
	(−0.528)	(−0.418)	
lnVillage water conservancy facilities			−0.002
			(−0.361)
_Cons	2.197 ***	1.598 ***	0.849 ***
	(5.481)	(4.168)	(3.565)
N	3472	3472	3472
r2	0.471	0.432	0.822
r2_a	0.469	0.430	0.822

Note: **, and *** indicate significance levels at 5%, and 1%, respectively.

**Table 6:** The heterogeneous results of the types of agricultural subsidies.

	(1)	(2)	(3)	(4)
	lnGrain Yield	lnGrain Yield	lnGrain Yield	lnGrain Yield
lnincome subsidy	0.539 ***			
	(30.445)			
lnsubsidy for superior varieties		0.485 ***		
		(30.330)		
lnsubsidies for agricultural supplies			0.321 ***	
			(4.999)	
lnsubsidies for purchasing agricultural machinery				0.377
				(1.146)
Household with party member	0.008	0.013	−0.032	0.754
	(0.177)	(0.158)	(−0.116)	(0.726)
Ethnicity	0.252 ***	0.335 ***	0.233	0.937
	(3.872)	(3.999)	(0.631)	(0.668)
Household labor force	−0.003	−0.025	0.003	−0.040
	(−0.159)	(−0.790)	(0.073)	(−0.105)
Cultivated area	0.007 ***	0.003 ***	0.007 **	0.059 *
	(10.372)	(5.652)	(2.086)	(2.131)
lnHousehold facilities of agricultural fixed assets	−0.036	−0.098	0.645 **	−0.526 *
	(−0.907)	(−1.309)	(2.036)	(−1.983)
lnWater, electricity, and irrigation costs for grain	0.369 ***	0.161 ***	0.570 **	−0.180
	(16.701)	(3.901)	(2.291)	(−0.352)
lnAnnual household net income	0.051 **	0.179 ***	0.062	−0.305
	(2.009)	(4.095)	(0.431)	(−0.595)
Terrain	−0.028	−0.048	0.306 **	0.624 *
	(−1.275)	(−1.412)	(2.257)	(1.941)
Village water conservancy facilities	0.000	0.000*	−0.000	0.000
	(0.702)	(1.702)	(−1.012)	(0.167)
_Cons	2.498 ***	3.394 ***	−3.789	11.358
	(5.353)	(4.023)	(−1.203)	(1.500)
N	2725	789	131	27
r2	0.455	0.675	0.467	0.712
r2_a	0.452	0.669	0.408	0.424

Note: *, **, and *** indicate significance levels at 10%, 5%, and 1%, respectively.

**Table 7:** Robustness test.

	(1)	(2)
	Use Data in 2016	Winsorize
lnAgricultural subsidy	0.525 ***	0.440 ***
	(37.157)	(27.266)
Gender	−0.015	−0.032
	(−0.258)	(−0.591)
Age	−0.002	−0.001
	(−1.455)	(−0.421)
Length of education	0.007	0.008
	(1.109)	(1.356)
Household with party member	0.014	0.021
	(0.366)	(0.596)
Ethnicity	0.253 ***	0.230 ***
	(4.429)	(4.341)
Household labor force	−0.003	−0.006
	(−0.240)	(−0.424)
Cultivated area	0.005 ***	0.017 ***
	(8.816)	(15.150)
lnHousehold facilities of agricultural fixed assets	−0.024	−0.031
	(−0.706)	(−0.666)
lnWater, electricity, and irrigation costs for grain	0.350 ***	0.325 ***
	(17.105)	(15.487)
lnAnnual household net income	0.071 ***	0.082 ***
	(3.516)	(4.083)
Terrain	−0.085 ***	−0.116 ***
	(−4.500)	(−6.910)
Village water conservancy facilities	−0.000	−0.000
	(−0.107)	(−0.446)
_Cons	2.106 ***	2.618 ***
	(5.325)	(5.505)
N	3423	3472
r2	0.467	0.492
r2_a	0.465	0.490

Note: *** indicate significance levels at 1%, respectively.

## Data Availability

The datasets used or analyzed during the present study are available from the corresponding authors on reasonable request.
